# Structure of Bovine Glycine *N*‑Acyltransferase Clarifies Its Catalytic Mechanism

**DOI:** 10.1021/acs.biochem.5c00315

**Published:** 2025-09-12

**Authors:** Ana C. Ebrecht, Christoffel P. S. Badenhorst, Uwe T. Bornscheuer, Randy J. Read, Diederik J. Opperman, Alberdina A. van Dijk

**Affiliations:** † Department of Microbiology and Biochemistry, 37702 University of the Free State, Bloemfontein 9301, South Africa; ‡ Institute of Biochemistry, Department of Biotechnology and Enzyme Catalysis, Greifswald University, Greifswald 17487, Germany; § Department of Haematology, Cambridge Institute for Medical Research, 2152 University of Cambridge, Cambridge CB2 1TN, United Kingdom

## Abstract

Glycine *N*-acyltransferase (GLYAT; EC 2.3.1.13, Accession ID: AAI12537) is a key enzyme in mammalian homeostasis that has been linked to several pathologies in humans, including cancer. Here we report the first crystal structure of a member of the GLYAT family, both in the apo form as well as bound to benzoyl-CoA. Binding of glycine could be inferred from an acetate molecule from the crystallization solution. A detailed analysis of its structure and the effects of mutations of key residues helped elucidate the catalytic mechanism, showing a general base-catalyzed reaction driven by a potential low-barrier hydrogen bond (LBHB) formed between the catalytic Glu-His dyad. This work will aid further studies of GLYAT and other members of the family.

## Graphical abstract

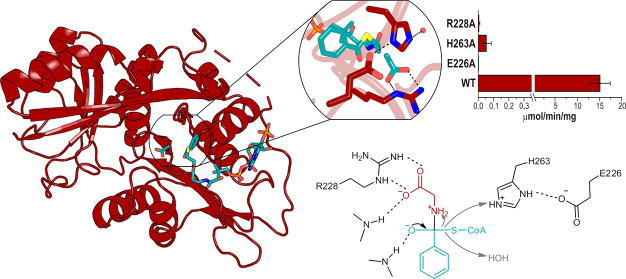

GLYAT participates in phase II detoxification through conjugation of benzoyl-CoA to glycine to form hippurate, one of the four most abundant metabolites in urine. This makes GLYAT essential to the homeostasis of hepatic and renal coenzyme A (CoASH), which would otherwise be sequestered as benzoyl-CoA.^1,2^ Recently, glycine conjugation has gained more awareness due to the excessive use of some products such as benzoate (as a preservative) and common drugs like aspirin.^3^ The metabolism of these everyday products exacerbates the dietary deficiency of glycine, a conditionally essential amino acid in humans, influencing the metabolism of not only glycine but also ATP and free CoASH, which participate in many metabolic pathways. Glycine deficiency is associated with pathologies like CASTOR (Coenzyme A Sequestration, Toxicity, and Redistribution) disorders,^4^ and its metabolic product, hippurate, has been identified as a biomarker for toluene exposure.^5,6^ In addition, polymorphism of the GLYAT gene has been linked to variation in musculoskeletal growth,^7^ and its expression is downregulated in liver and breast cancer cells.^8,9^

To gain insight into the catalytic mechanism, orthologous GLYAT from *Bos taurus* (bGLYAT) was recombinantly expressed and purified (Figure S1). The protein was crystallized both alone and in the presence of either glycine or benzoyl-CoA. The structures, to resolutions of 1.25–1.65 Å, were solved in parallel by both molecular replacement (MR) using RosseTTAFold^10^ and single-wavelength anomalous diffraction phasing based on the intrinsic sulfur atoms (S-SAD) (Table S2). The apoenzyme structures present two copies in the asymmetric unit (ASU), while only one copy is present in the structure solved with benzoyl-CoA. Size-exclusion chromatography confirmed that bGLYAT is a monomer in solution (Figure S1).

Although we failed to obtain bGLYAT crystals in complex with glycine, the electron density for an acetate molecule from the crystallization solution was detected and interpreted in the putative glycine binding site (Figure 1 and Figure S2).

**Figure 1:**
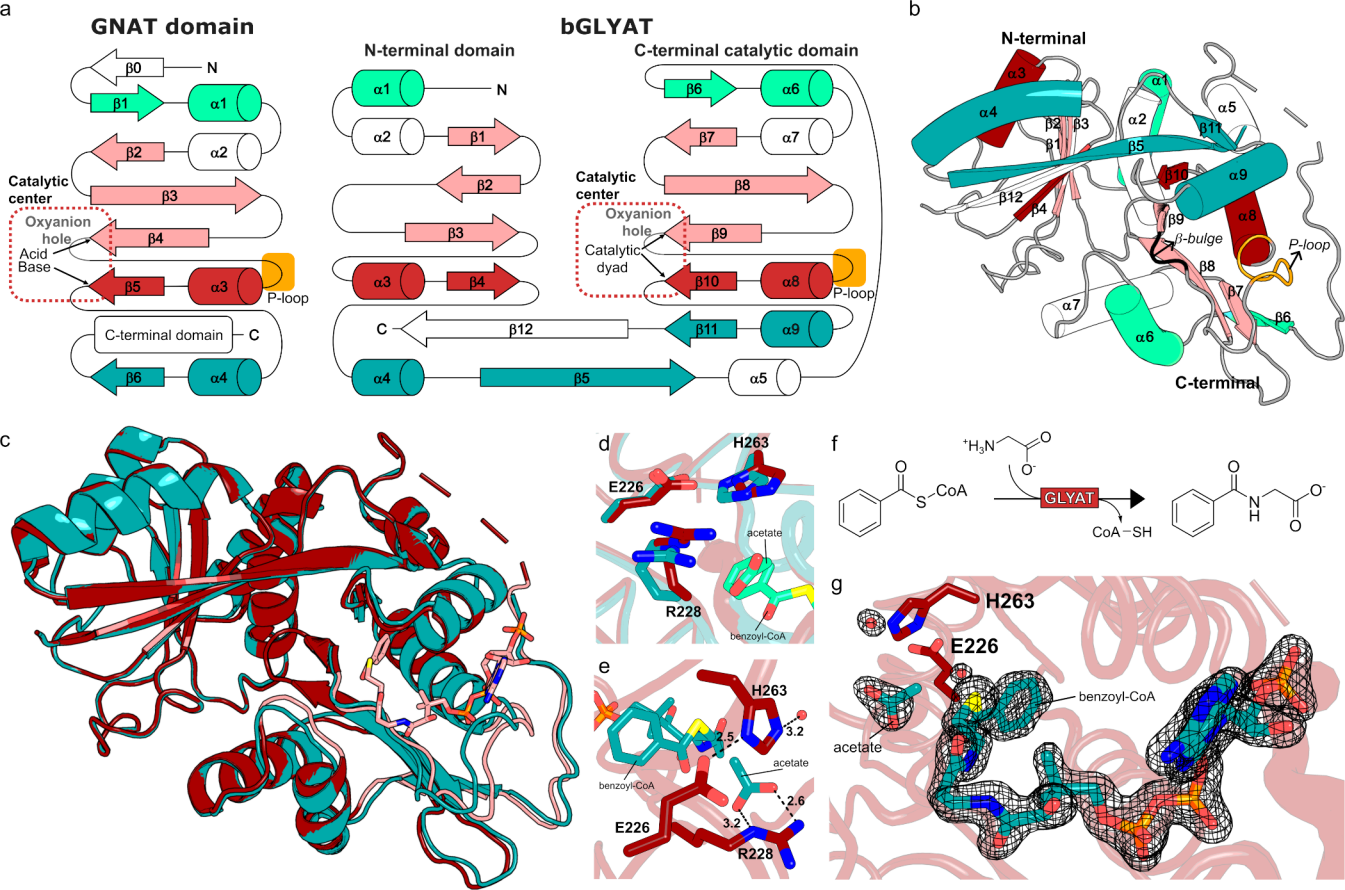
(a) General GNAT vs bGLYAT topology. (b) Overall view of the bGLYAT fold. (c) Comparison of the overall GLYAT structures with (red, 7PK0) and without (teal, 7PK2_B) benzoyl-CoA (pink). Changes in the structure upon substrate binding are highlighted in pink. (d) Movement of the residues in the catalytic site after binding of benzoyl-CoA. 7PK2_B is colored teal and 7PK0 is colored red. (e) Binding of the acetate molecule (teal) in the putative binding site of glycine. (f) Scheme of the reaction catalyzed by GLYAT. (g) Omit map 2Fo-Fc (σ = 1.5 Å) for benzoyl-CoA, acetate, and the putative catalytic water (red sphere).

GLYAT belongs to the Gcn5-related *N*-acetyltransferase (GNAT) superfamily.^1^ Despite the low sequence identity among members, the catalytic domain’s topology is conserved. However, members of the family have diverged to adopt additional structural elements (Figure 1a). These structural differences significantly alter the substrate binding site’s architecture and the mechanisms for deprotonation of the primary amine to promote nucleophilic attack.^11^ The bGLYAT structure comprises two distinct domains with almost identical topology: a mixed open central β-sheet with helical crossovers, typical of the GNAT fold^11^ (Figure 1a,b). The domains are linked by a disordered loop that showed low to no density for residues 144–157. The N-terminal domain and C-terminal catalytic domain are related to each other by a pseudo-2-fold axis that is not altered by the binding of benzoyl-CoA (Figure 1c). The C-terminal strand β12 extends and forms part of the N-terminal central β-sheet, while the N-terminal strand β5 extends to the C-terminal catalytic domain and positions antiparallel to β10 and β11, generating backbone contacts that stabilize the structure. A similar type of fold has been described for the human *N*-myristoyl transferase, although the catalytic site is found there in the N-terminal domain^12^ (Figure S3).

In the catalytic domain, the topological switch point is located between strands β9 and β10, forming a V-shaped cleft that accommodates the benzoyl-CoA. The acyl donor adopts a C-shaped orientation and interacts with the enzyme via hydrophobic contacts and direct and water-mediated hydrogen bonds (Figure S4), similar to what has been reported for other GNAT-CoA structures.^11^

The benzoyl moiety is buried in a hydrophobic pocket formed by β9-10, α8, and loop α6-7, where it is stabilized by hydrophobic and van der Waals interactions with Met227, Met271 Ile246, and Ser262. In the same pocket, an acetate anion is found with its methyl group (where the amino group of glycine would be attached) pointing toward the thioester bond of the acyl donor benzoyl-CoA (Figure 1). A glycine molecule was modeled in the acetate position in two different C–Cα torsion angles showing the close proximity to the catalytic dyad and acyl donor (Figure S2). The carboxyl group of the acetate interacts with NH_2_ and the NE of Arg228. In the apoenzyme, this residue coordinates a sulfate or malonate ion from the crystallization solution (Figure S5).

Two characteristic GNAT features are present in the bGLYAT structure: the β-bulge, creating the oxyanion hole that stabilizes the tetrahedral intermediate during the reaction, and the “P-loop”, interacting with the pyrophosphate moiety of the CoA.^11^ The structural fold resembles a βαβ Rossmann fold, usually found in nucleotide diphosphate binding proteins. However, the characteristic glycine motif (Gly-X-Gly), which allows the hairpin turn and interacts with the diphosphate group, is replaced by Ala-X-Gly in the bGLYAT structure.

His263 is positioned near the substrates and forms a short-distance hydrogen bond (HB) with Glu226 (the Glu226 OE2–His263 ND1 distance is ∼2.5 Å). This short distance between the residues is characteristic of a potential low-barrier hydrogen bond (LBHB), compared to typical hydrogen bonds with distances between heteroatoms of 2.8–3 Å.^13^ This type of bond has previously been described for another GNAT member, the aminoglycoside *N*3-acetyltransferase–VIa, where a LBHB is formed between a Glu and His.^14^ A LBHB would stabilize the active site and not only position the His but also facilitate catalysis by increasing the basicity of its NE2 by increasing the p*K*_a_, driving the abstraction of a proton from glycine, similar to what occurs with the aminoglycoside *N*3-acetyltransferase–VIa.

The mutation of the Glu residue in bovine^15^ (E226Q) and human^16^ (E227Q) GLYAT to Gln significantly decreased the enzymatic activity (more than 20-fold), supporting the key role of this acid residue for catalysis. To further evaluate the importance of the catalytic dyad and to investigate the effect of hydrogen bonding between the residues, we generated the variants E226A, E226D, and H263A. Whereas mutant E226A did not have detectable activity, mutants E226D and H263A had a 300- and 60-fold lower activity compared to the wild-type, respectively (Figure 2 and Figure S6). In addition, all these enzymes showed an increase in activity at pH 9.0 (5–20 times) compared to their activities at pH 7.5, supporting the need of an environment that increased the basicity of His263 and approaching the p*K*_a_ of glycine’s amino group, similar to what was observed for the mutant E226Q.^15^ The structures show the coordination of water molecules in the catalytic pocket that interact with the catalytic dyad and the molecules bound to the putative glycine site. These interactions could also influence the p*K*_a_ of the glycine amino group, making it susceptible to deprotonation at pH 9.0, which would explain the increase in activity for the H263A variant (Figure 2 and Figure S6).

**Figure 2:**
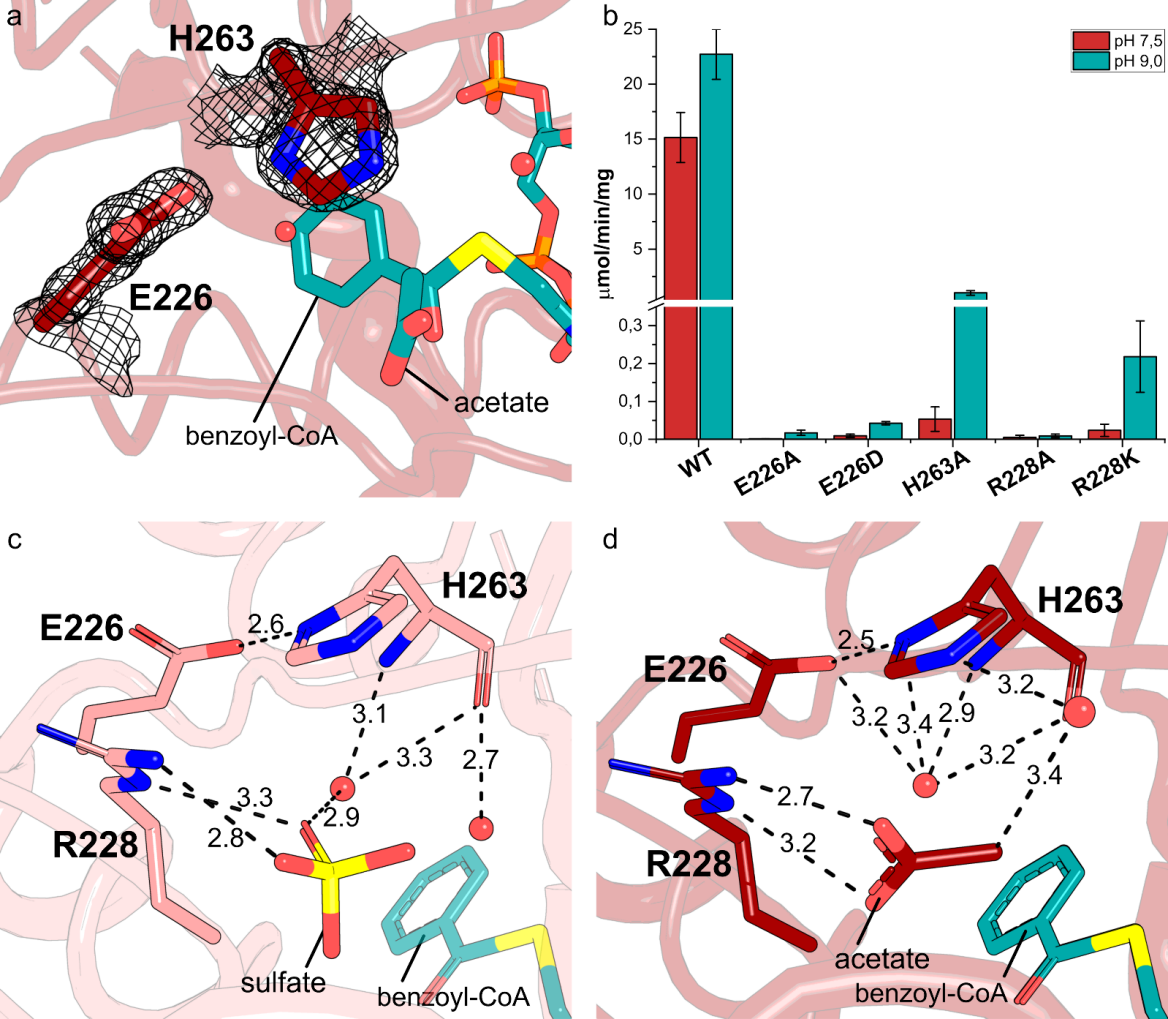
(a) Omit map 2Fo-Fc (σ = 1.5 Å) for the catalytic-dyad Glu226-H263. (b) Comparison of the enzymatic activity of bGLYAT and its mutants. (c) Binding of a sulfate anion in the putative glycine binding site for structure 7PK1. Benzoyl-CoA from the structure 7PK0 is modeled in the catalytic site for reference. (d) Binding of an acetate anion in the putative glycine binding site for the structure 7PK0.

Based on this analysis of the structure, we reevaluated the mechanism proposed previously.^15^ The enzyme follows a general base-catalyzed ternary complex mechanism coordinated by the catalytic dyad Glu226-His263 (Scheme 1). The structure also shows that His263 coordinates a water molecule; thus, the deprotonation of glycine could occur directly, or it is water mediated, as it has been observed for some GNAT superfamily members.^11^ This is in agreement with the observation that H263A shows activity, albeit severely reduced, and the activity increased with the increase of the pH in the reaction. Additionally, the presence of several water molecules in the catalytic pocket suggests that glycine deprotonation could occur via a potential water bridge with the carboxyl group of Glu226 in the absence of His263, as has been observed in some GNAT family members that utilize a water-residue network for deprotonation.^17^

**Scheme 1:**
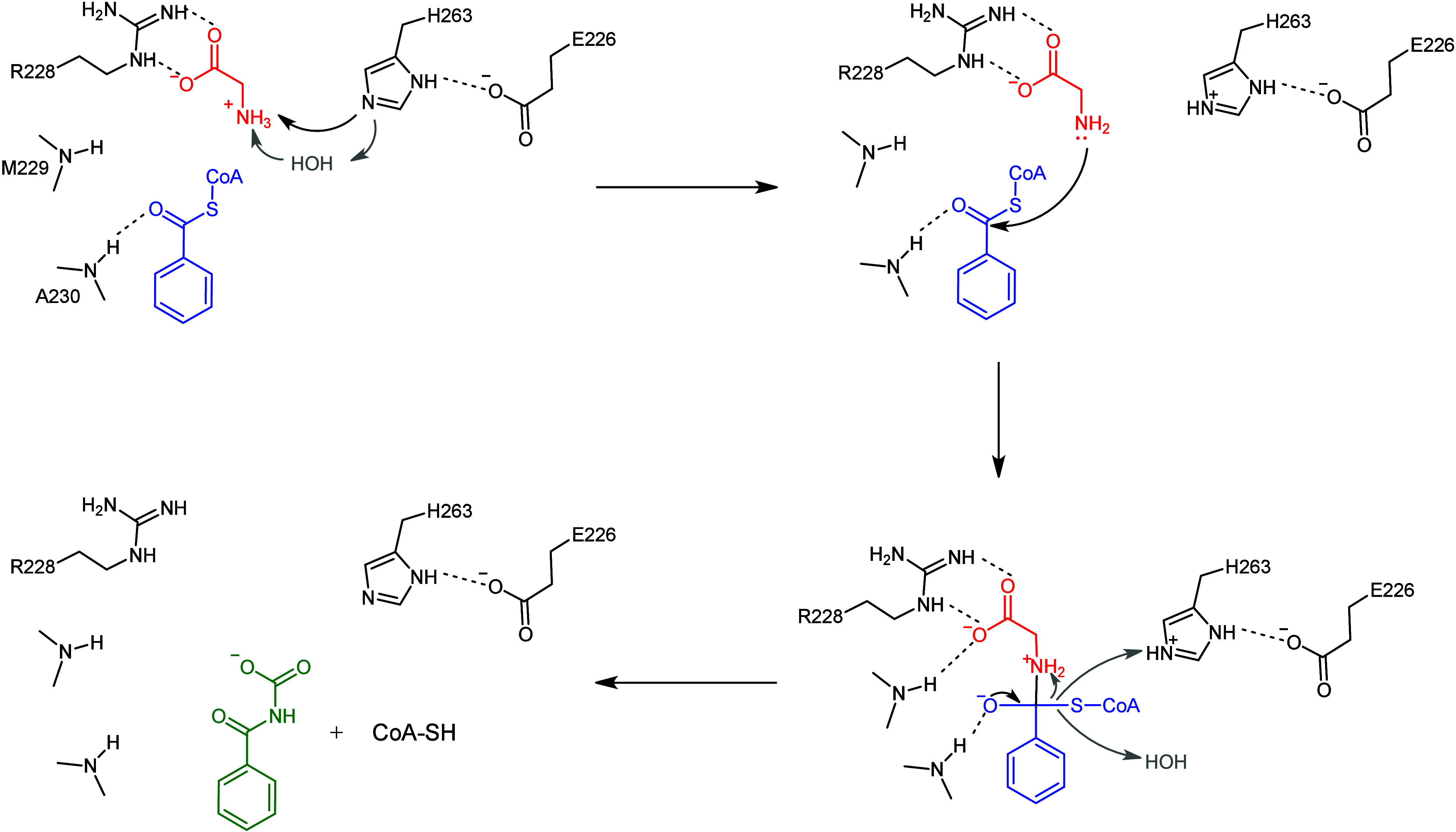
Proposed Catalytic Mechanism of bGLYAT

The conserved Arg228 coordinates the carboxyl group of the acyl donor via a salt bridge. This interaction favors the activation of the substrate by polarizing the glycine molecule and therefore facilitating the removal of the proton and preventing the reprotonation before the nucleophilic attack occurs. The mutation of this residue to an Ala (R228A) abolished the activity of the enzyme, while the mutation R228K decreased the activity (100 times) and the apparent affinity for glycine but not benzoyl-CoA (Figure 2 and Figure S6).

Interestingly, the enzyme can use other amino acids (including Ala, Asn, Gln, and Glu) as acyl acceptors but with poor efficiency.^1^ Although these alternative acceptors are larger amino acids, we hypothesize that the effect on the reduced activity is probably due to a loss of the salt bridge polarization effect and not due to steric hindrance.

After deprotonation, nucleophilic attack by the amino group of the glycine on the thioester carbonyl group of acyl-CoA takes place. Arg228 was found in different conformations in the apo structure, depicting a certain degree of mobility (Figures 1d and S2d). We hypothesize that this residue could also play a crucial role in positioning the substrate relative to the His263 for deprotonation and subsequently repositioning the activated glycine for nucleophilic attack.

Met229-Ala230 form the β-bulge in β9; the amide groups of these residues are directed toward the carbonyl oxygen of the benzoyl-CoA. The oxyanion formed polarizes the C–S bond and stabilizes the zwitterionic tetrahedral intermediate.

Finally, the intermediate collapses into free CoASH and acyl-glycine products. With the exception of the now protonated His263, no obvious residue is present as a general acid candidate to protonate the thiolate. The protonation could, however, occur during molecular rearrangement of the intermediate through an intramolecular proton transfer from the amide group. As electron density for a number of water molecules was also observed within the active site, protonation of the leaving group from the solvent cannot be excluded (Scheme 1).

Due to its physiological relevance, human GLYAT has recently received considerable attention. Some groups have reported genetic polymorphisms in the GLYAT gene, and in vitro studies of the prevalent variants showed some of the mutations affect the enzymatic activity.^16,18,19^ GLYAT is, however, highly conserved in mammals, and the bovine and human enzymes share similar catalytic efficiencies and substrate specificities.^15^ Not surprisingly, these two enzymes share an 83% sequence similarity (76% pairwise identity), and the residues involved in catalysis are conserved (Figure S7). The bGLYAT structure, and the insights obtained from the substrates’ binding, can therefore contribute to the rational design of experiments to understand how these changes impact the efficiency of GLYAT and contribute to its pharmacogenetic consequences.

The overall structure is novel to the superfamily, with an N-terminal GNAT domain that does not have a clear function. In other members of the family, such as GLYAT-like2, it has been implicated in allosteric regulation of the enzyme’s activity via acetylation.^20^ The GLYAT sequence presents several putative post-translational modification sites for acetylation and phosphorylation (Figure S7). Interestingly, this enzyme has been related to kinase pathways, such as PI3K/AKT in human breast cancer cells.^11^ All these require more in-depth study of GLYAT and its multiple roles in human metabolism, which is beyond the scope of this work.

For many years, the importance of glycine *N*-acyltransferase has been underestimated, and its study has thus been neglected. However, there is increasing evidence of the significance of this enzyme for the proper functioning of the human organism. Indeed, until recently, no defect in this gene has ever been reported, supporting the essential role of glycine conjugation in the metabolic pathway.^19,21^ Recently, downregulation of GLYAT has been proposed as a biomarker and possible target for breast cancer therapy.^11^ The crucial value of this protein requires an in-depth understanding of its structure and enzymatic mechanism. The crystal structure of the first member of the family is a step forward toward this goal.

## Supplementary Material



## Notes

Experimental procedures, crystallographic data, structural and sequence alignment, analysis of binding of substrates, and kinetic analysis of mutants (PDF)
