# Supplementary material for: Structure of Bovine Glycine N‑Acyltransferase Clarifies Its Catalytic Mechanism
Source: Biochemistry. 2025 Sep 12;64(19):4050–4. doi: 10.1021/acs.biochem.5c00315 (PMC12509317; doi:10.1021/acs.biochem.5c00315)
Supplement: Supplementary file 1 [file bi5c00315_si_001.pdf]

Supporting Information

Structure of bovine glycine *N*-acyltransferase clarifies its catalytic mechanism

Ana C. Ebrecht[1], Christoffel P.S. Badenhorst[2], Uwe T. Bornscheuer[2], Randy J. Read[3], Diederik J. Opperman[1*], Alberdina A. van Dijk[1*]

[1]*Department of Microbiology and Biochemistry, University of the Free State, Bloemfontein, 9301, South Africa*

[2]*Institute of Biochemistry, Department of Biotechnology and Enzyme Catalysis, Greifswald University, 17487 Germany*

[3]*Department of Haematology, Cambridge Institute for Medical Research, University of Cambridge, CB2 1TN, United Kingdom*

*Corresponding authors:

OpperDJ@ufs.ac.za

VanDijkAA@ufs.ac.za

Experimental Procedures

Cloning and expression. The open reading frame (ORF) coding for *Bos taurus* glycine *N*-acyltransferase (bGLYAT, NP_803479) was fused to the small ubiquitin modifying protein (SUMO) tag (N-terminal), separated by a TEV protease cleavage site to facilitate cleavage of the tag (SUMO-TEV-bGLYAT). The construct was synthesized and cloned into pET-28a(+), using the NdeI and XhoI sites, by GenScript. bGLYAT mutants were obtained using the megaprimer protocol[1] using primers from Table S1.

Table S1. Primers used for introducing mutations in GLYAT

Mutation	Primer	T_m (°C)
E226A	CTG CCA TCC GCA T**CG C**TC CCG TCT G	67.4
E226D	CCT GCC ATC CGC AT**A TC**T CCC GTC TGG TC	66.6
R228A	GTG CCT GCC AT**C GC**C ATC TCT CCC GTC TG	68.7
R228K	GTG CCT GCC AT**T TT**C ATC TCT CCC GTC	63.2
H263A	GTT CTT GGG GTC CAC **CGC** AGA GTA CAC AGG	66.4
Universal reverse primer	GTT CCT GCT GCA AGG TGC CCA GAT GCT G	66.8

PCRs (50 µL) consisted of 1X KOD Hot Start Polymerase buffer, 1.5 mM $MgSO_4$, 0.2 mM (each) deoxyribonucleotide triphosphates (dNTPs), 0.5 U KOD Hot Start polymerase (Novagen), 0.4 ng·mL^{-1} template DNA and 0.1 µM of both forward and reverse primers. PCR conditions consisted of an initial denaturation step (95 °C, 2 min), followed by 6 cycles of denaturation at 95 °C (20 s), annealing at 60 °C (10 s) and elongation at 70 °C (13 s) followed by 30 cycles of denaturation at 95 °C (20 s) and annealing and extension of the megaprimer at 70 °C (3 min) with a final extension of 12 min at 70 °C.

Constructs for the expression of TEV protease and Benzonase were generously donated by Prof. F. von Delft (Oxford University).

Enzymes were expressed using *Escherichia coli* BL21-Gold(DE3) cells (Stratagene). Cultures were grown in the auto-induction medium ZYM-5052 for 48 h at 25 °C[2].

Protein purification. For purification of bGLYAT, Benzonase, and TEV protease, cells were harvested by centrifugation (5000 x *g*, 10 min, 4 °C). The pellets were resuspended (0.2 $g_{wet\ weight}$ mL^{-1}) in BugBuster protein extraction reagent (Novagen) containing 30 U mL^{-1} lysozyme

(Fluka) and 3 μg mL^{-1} Benzonase and incubated at room temperature for 15 min. The insoluble material was removed by centrifugation (20 min, 20,000 x *g,* 4 °C) and the clarified lysate was loaded onto a 5 mL HisTrap FF column (GE Healthcare), previously equilibrated with buffer A (25 mM Tris-HCl, 40 mM imidazole, and 500 mM NaCl, pH 8.0). The column was washed with 10 column volumes of buffer A, and the protein was eluted with a linear gradient of imidazole (40 – 300 mM) using an ÄKTA pure automated system (GE Healthcare). Fractions containing the target protein were pooled, concentrated through ultrafiltration (Amicon Ultra-10 kDa MWCO (Merck)), and desalted by size exclusion chromatography (SEC) using PD-10 columns (GE Healthcare) pre-equilibrated with buffer B (25 mM Tris-HCl and 50 mM NaCl, pH 8.0). Cleavage of the SUMO tag from the bGLYAT was performed by the addition of TEV protease (1 μg TEV/100 μg fusion protein) to the desalted fraction and overnight incubation at 4 °C. The cleaved protein was passed through a 5 mL HisTrap FF column, equilibrated with buffer A, and the flow-through, containing the bGLYAT, was collected and concentrated as described above. The concentrated pool was used to perform SEC using Sephacryl S200HR (XK 26/100 column, GE Healthcare) equilibrated with buffer C (10 mM Tris-HCl, pH 8.0). The eluted bGLYAT was pooled and concentrated.

Benzonase and TEV protease were kept at -20 °C, after the addition of glycerol to a final concentration of 50%.

For determination of the quaternary structure, analytical size exclusion chromatography was performed using a Yarra 3 μm SEC-3000 column (300 mm x 4.6 mm).

Protein concentration and activity determination. The protein concentration was determined by the BCA assay (Pierce) using bovine serum albumin as standard. The activity of bGLYAT was analyzed by following the reduction of DTNB [5,5'-dithiobis(2-nitrobenzoic acid)] (Merck) at 412 nm using a Multiskan SkyHigh Microplate Spectrophotometer. Reactions were performed as described elsewhere[3] ($\varepsilon = 13.6\ mM^{-1}cm^{-1}$, 50 mM TrisHCl pH 8.0, T = 25 °C). Protein purity was analyzed by SDS-PAGE.

Crystallization, data collection, and structure determination. The purified bGLYAT was concentrated to 6 and 8 mg mL^{-1}. Crystals were grown by the sitting drop vapor diffusion

method, by mixing 1 µL of protein and 1 µL of the reservoir solution (0.2 M sodium malonate, 20% w/v polyethylene glycol 3,350, pH 5.0, or 1.5 M ammonium sulfate, 0.1 M sodium acetate, pH 4.6). For crystals with benzoyl-CoA, the enzyme was crystallized in a 5-fold molar excess of the substrate (1 M sodium acetate, 30% v/v polyethylene glycol 300, pH 4.5). Plates were incubated at 16 °C. Crystals were soaked in a reservoir solution containing 20% v/v polyethylene glycol 400 before cryocooling. X-ray diffraction data were collected at Diamond Synchrotron (UK) on beamline I03 (0.9762 Å) at 93 K. Data were processed using XDS[4].

The apoenzyme crystallized in either space group $P2_12_12_1$ or $P6_5$ with two molecules in the asymmetric unit (ASU) that are almost identical in each crystal form (Cα RMSD of 0.372 and 0.479 Å, respectively). The bGLYAT-benzoyl-CoA complex crystallized in space group $P4_32_12$ with one molecule in the ASU and a related packing to the orthorhombic crystal form.

De novo phasing through single-wavelength anomalous dispersion of S atoms (S-SAD) were performed on crystals without substrate. Data were collected on beamline I23 (Diamond Synchrotron) at 4.5 keV (2.755 Å) and processed using XDS through xia2i[5]. Four datasets were merged using XSCALE[6]. A good anomalous signal was detected at 2.2 Å. SHELXD[7] and CRANK2[8] were used for structure solution and initial model building.

Several attempts to use very distant homologs for molecular replacement (MR) failed. Instead, five bGLYAT models were generated *ab initio* with RosseTTAFold[9]. The models were superimposed into an ensemble to use as a search model for MR. A trimmed ensemble of the models was used for molecular replacement using PHASER[10]. Iterative cycles of model building in COOT[11] and refinement using Refmac[12] were performed. Figures were prepared using PyMOL.

Table S2. X-ray data collection and refinement parameters

Data collection	S-SAD	Native1	Native2	Benzoyl-CoA
Beamline DSL	I23	I03	I03	I03
Wavelength (Å)	2.755	0.9762	0.9762	0.9762
Space group	P 21 21 21	P 21 21 21	P 65	P43 21 2
Cell dimensions a/b/c (Å)	63.5/64.2/135.8	63.1/63.6/135.5	96.2/96.2/144.6	63.8/63.8/135.3
α/β/γ (°)	90/90/90	90/90/90	90/90/120	90/90/90
Resolution (Å)	58.09-1.80 (1.83-1.80)	37.37-1.25 (1.27-1.25)	48.09-1.65 (1.68-1.65)	46.44-1.50 (1.53-1.50)
Unique reflections	48496	146849	99910	45806
Completeness (anomalous) (%)	93 (86)	97.5	96.7	100
I/sig(I)	11.5	16.2	18.7	8.1
Multiplicity (anomalous)	6.5 (3.6)	6.8	7.9	12.5
R_{meas} (outer shell)	0.089 (1.0)	0.048 (2.3)	0.049 (1.4)	0.108 (2.4)
CC1/2 (CCano)	1.0 (0.4)	1.0	1.0	1.0
Molecules in ASU	2	2	2	1
**Refinement**				
R_{work}/R_{free}		0.177/0.208	0.180/0.203	0.181/0.211
Number of residues modelled		568	575	284
Average B-factor, all atoms ($Å^2$)		23.0	34.0	31.0
RMS Bond lengths		0.014	0.016	0.015
RMS Bond angles		1.79	1.87	1.77
Ramachandran outliers (%)		0	0	0
MolProbity score		1.55	1.55	1.20
Poor rotamers (%)		0.53	0.92	1.5
Clash score, all atoms		3.88	3.06	3.35
PDB ID		7PK2	7PK1	7PK0

Values in parenthesis describe the outer shell.

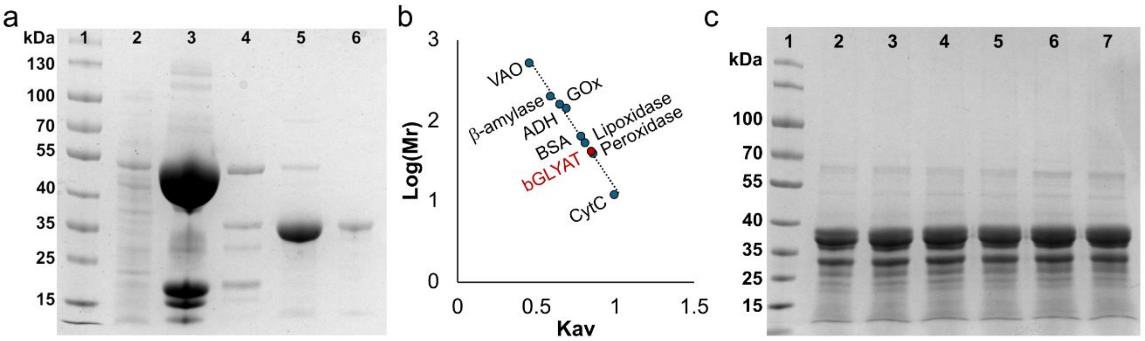

Figure S1. Purification of the bovine GLYAT and mutants. The enzyme was expressed as a fusion to a His-tagged SUMO peptide to improve solubility. High levels of soluble expression were obtained for the SUMO-GLYAT fusion. After cleavage with TEV protease, one glycine residue was added to the N-terminus of bGLYAT. An active bGLYAT was purified to near homogeneity, with an estimated yield of 10 mg per litre of culture. (a) SDS-PAGE for the analysis of expression and purification of bGLYAT. **Lane 1**: molecular weight marker, **lane 2**: crude extract loaded onto IMAC column, **lane 3**: purified SUMO-GLYAT fusion, **lane 4**: TEV protease cleavage of the fusion protein, **lane 5**: purified bGLYAT after second IMAC, **lane 6**: purified bGLYAT after size exclusion chromatography. (b) Analysis of quaternary structure of bGLYAT by analytical size exclusion chromatography was performed using a Yarra 3 µm SEC-3000 column (300 mm x 4.6 mm). A standard curve was generated with known molecular proteins. **VAO**: vanillyl-alcohol oxidase (520 kDa), **β-amylase** (200 kDa), **GOx**: glucose oxidase (160 kDa), **ADH**: alcohol dehydrogenase (147 kDa), **BSA**: bovine serum albumin (66 kDa), **lipoxidase** (54 kDa), **peroxidase** (40 kDa), **CytC**: cytochrome c (12 kDa). (c) SDS-PAGE for the purified mutants. **Lane 1**: molecular weight marker. **Lane 2**: bGLYAT wild-type, **lane 3**: bGLYAT E226A, **lane 4**: bGLYAT E226D, **lane 5**: bGLYAT R228A, **lane 6**: bGLYAT R228K, **lane 7**: bGLYAT H263A.

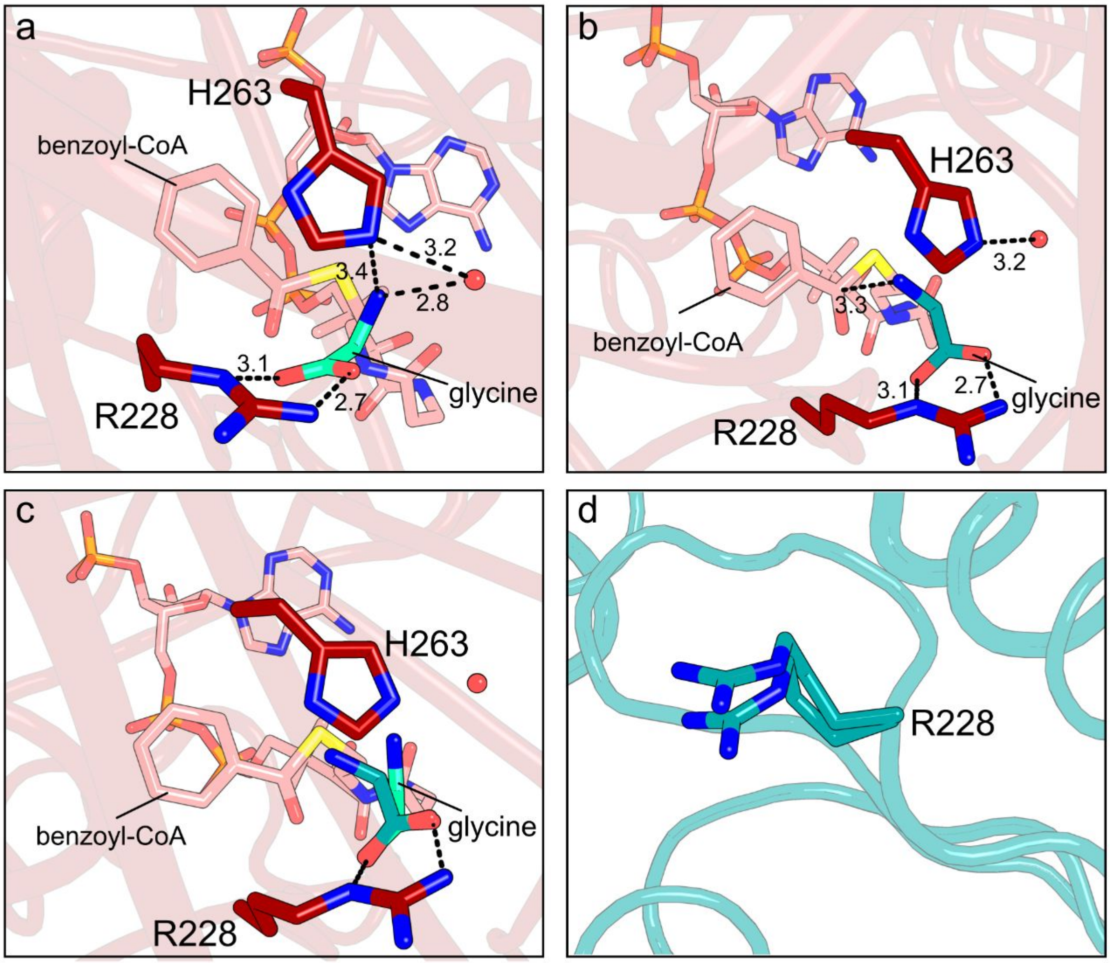

Figure S2. Binding of the acyl acceptor to the active site of bGLYAT. A glycine molecule (teal) was modelled in the position of the acetate bound to Arg228 in two different positions. (a) The amino group is pointing towards the putative catalytic water. The catalytic dyad (Glu226-His263) is shown pointing toward the substrates and all the distances are displayed. (b) The second conformation pointing towards the acyl donor is shown. The distance to the carbonyl thiol group is shown with dashed lines. (c) Superposition of the two conformations predicted for the glycine. The molecule is shown bound to the Arg228. (d) Different conformations of Arg228 found in one of the copies of the ASU in the apoenzyme structure.

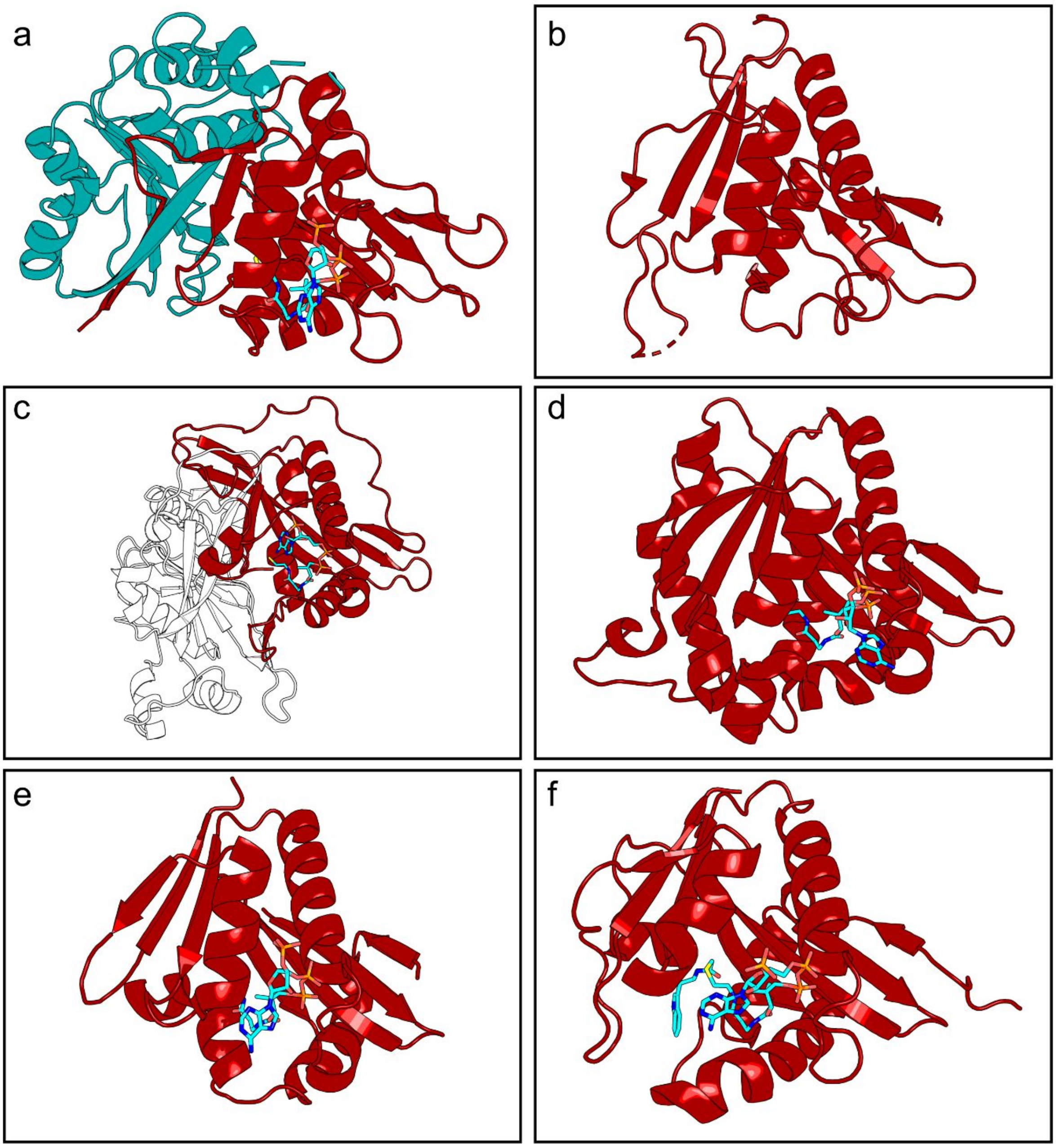

Figure S3. Structural comparison of bGLYAT to other GNAT enzymes. (a) bGLYAT structure displaying the substrate benzoyl-CoA in cyan. (b) Human *N*-α-acetyltransferase 20 (PDB ID 6VP9, chain A, Cα RMSD 1.5 Å). (c) Human *N*-myristoyl transferase structure displaying the CoASH molecule in cyan (PDB ID 6QRM, Cα RMSD 1.7 Å). (d) *Drosophila melanogaster* dopamine *N*-acetyltransferase in complex with acetyl-CoA (3TE4, Cα RMSD 1.7 Å). (e) *Sulfolobus solfataricus* protein acetyltransferase (3F8K, Cα RMSD 1.3 Å). (f) *Ovis aries* serotonin *N*-acetyltransferase in complex with the bisubstrate analogue CoA-S-acetyl tryptamine (1CJW, Cα RMSD 1.6 Å).

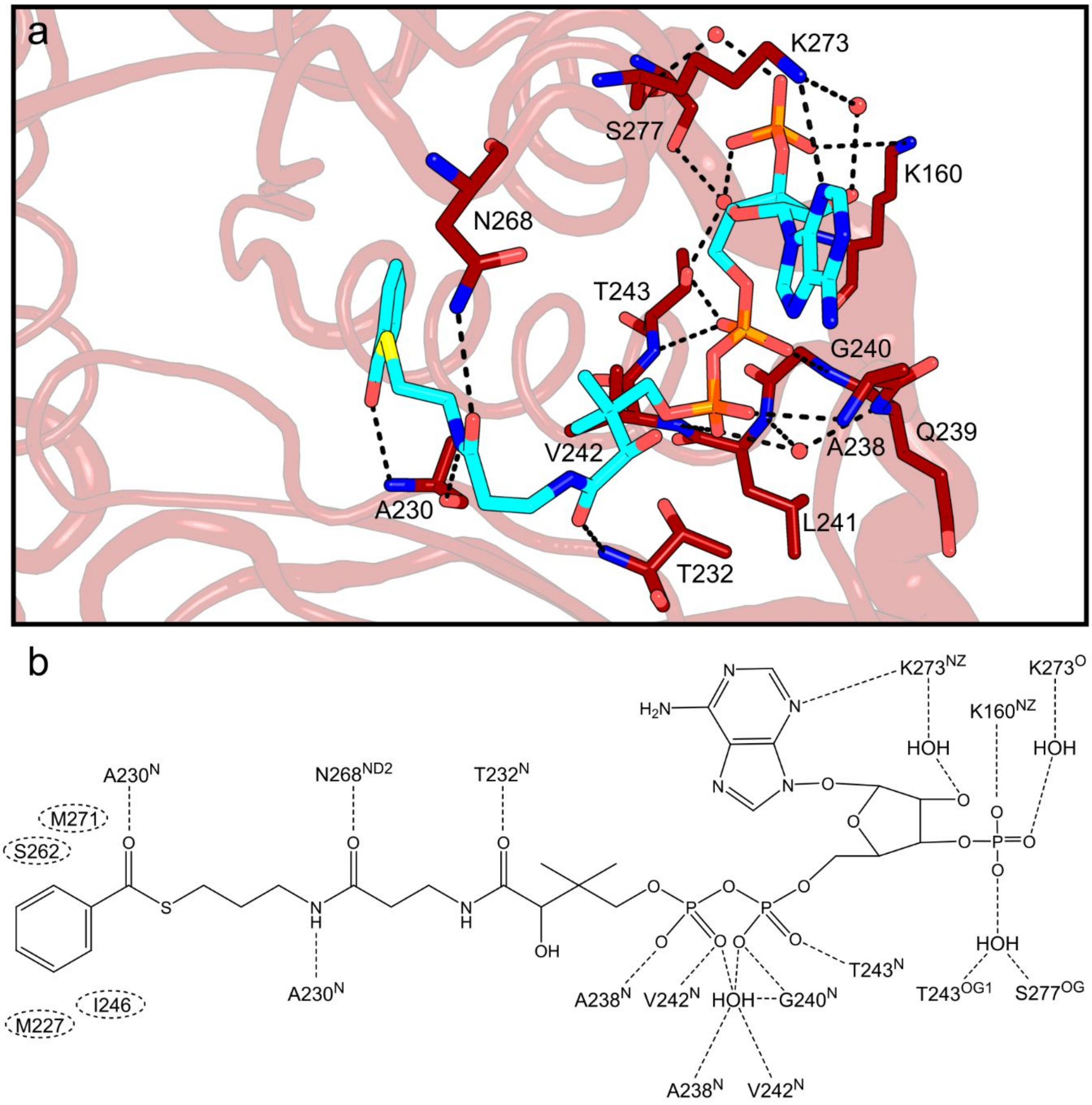

Figure S4. Interaction of benzoyl-CoA with the bGLYAT residues. (a) Residues from the active site interacting with the acyl donor are displayed. Water molecules are shown as red spheres and hydrogen bonds as black dashed lines. (b) Structural scheme of the benzoyl-CoA interactions with bGLYAT. Dashed lines indicate hydrogen bonds. Dashed circles show residues stabilizing the benzoyl ring through van der Waals and hydrophobic interactions.

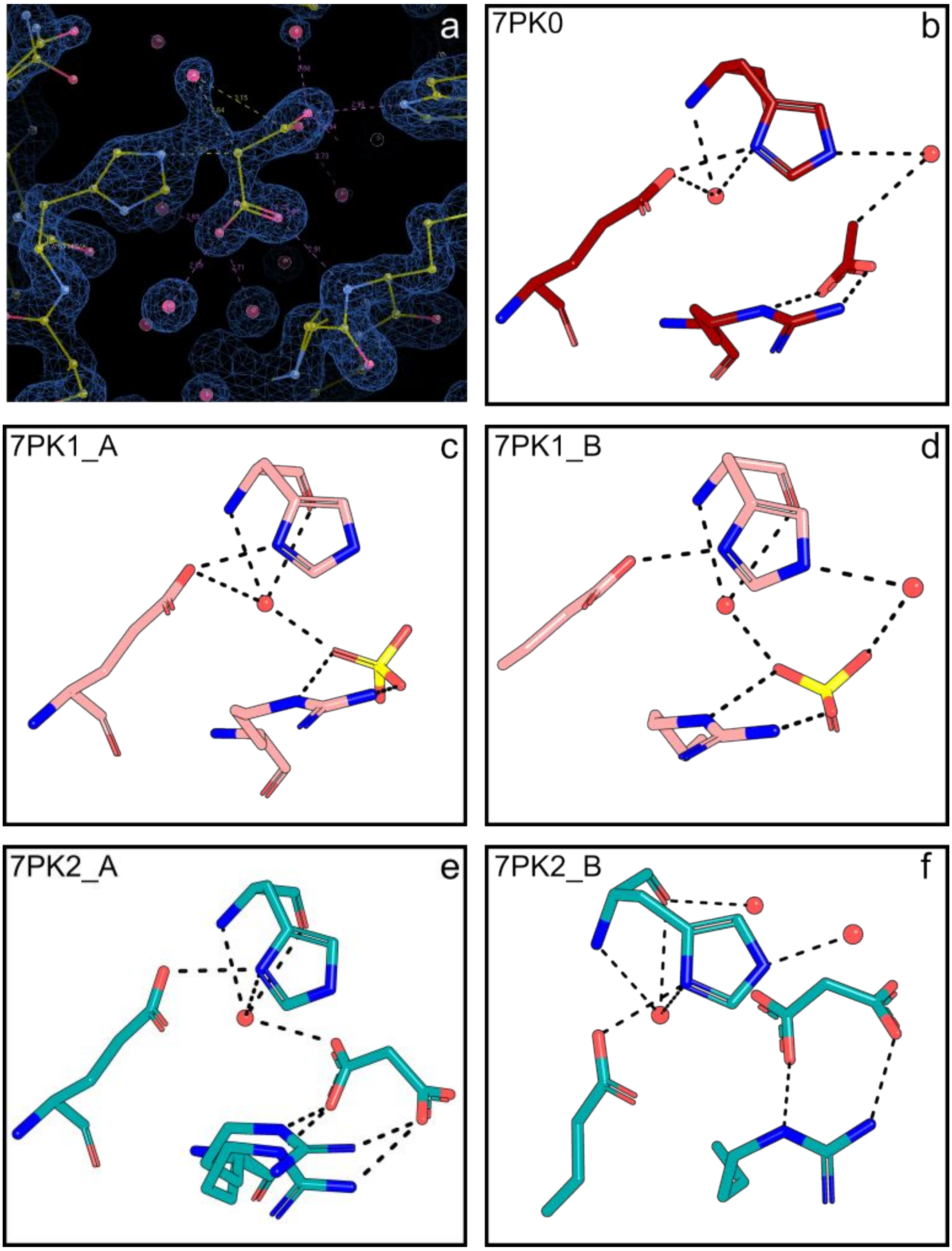

Figure S5. Catalytic pocket of bGLYAT. (a) Malonate adduct observed in the apoenzyme (PDB ID: 7PK2). (b) Binding of acetate molecule in the putative glycine binding site for the structure with benzoyl-CoA (PDB ID: 7PK0). (c-d) Binding of sulfate molecule in the putative glycine binding site for the structure of the apoenzyme (PDB ID: 7PK1). (e-f) Binding of malonate molecule in the putative glycine binding site for the structure of the apoenzyme (PDB ID: 7PK2). The residues Glu226, Arg228, and His263 are shown and the interactions shorter than 3.5 Å are shown as dashed lines.

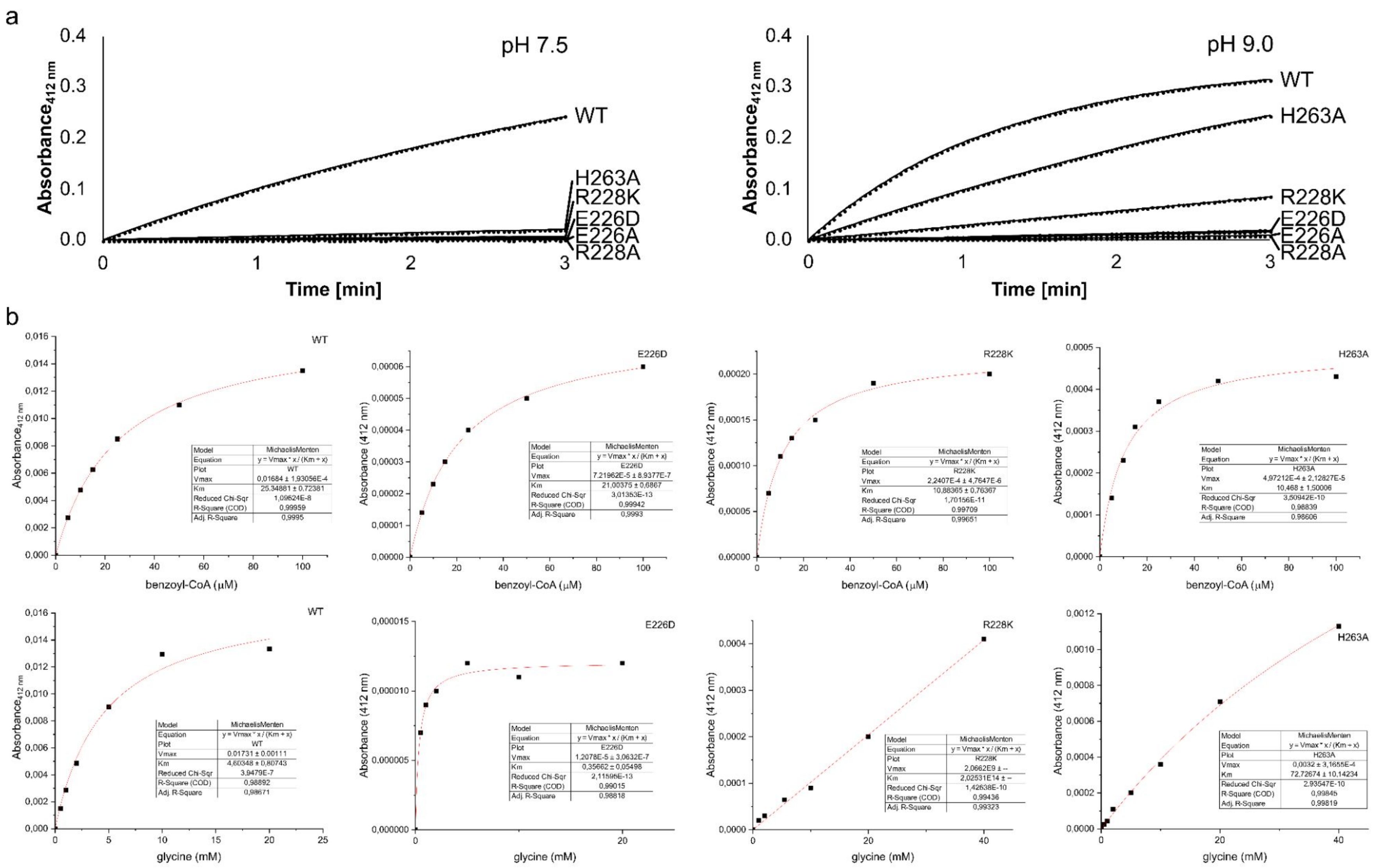

Figure S6. Enzymatic activity of bGLYAT and mutants. (a) Comparison of activity of WT bGLYAT and its mutants at pH 7.5 and 9.0. Reaction conditions: [enzyme] = 1 µg (WT) or 5 µg (mutants); [Tris-HCl] = 50 mM, pH 7.5 or 9.0; [glycine] = 20 mM; [benzoyl-CoA] = 100 µM; T = 25 °C. (b) Saturation curves for the substrates. Top: curves with different concentrations of benzoyl-CoA. Reaction conditions: [enzyme] = 5 µg/mL (WT) or 25 µg/mL (mutants); [Tris-HCl] = 50 mM, pH 7.5; [glycine] = 20 mM; T = 25 °C. Bottom: curves with different concentrations of glycine. Reaction conditions: [enzyme] = 1 µg (WT) or 5 µg (mutants); [Tris-HCl] = 50 mM, pH 7.5; [benzoyl-CoA] = 100 µM; T = 25 °C.

Table S3. Kinetic parameters for reactions at pH 7.5

**Enzyme**	***K*$_M$**		***k*$_{cat}$**
	**Glycine (mM)**	**Benzoyl-CoA (μM)**	**(min^{-1})**
**WT**	4.6	25.3	5048
**E226D**	0.4	7.3	3
**R228K**	>> 4.6	10.9	8
**H263A**	>> 4.6	10.5	18

* k_{cat} calculated with reaction conditions: [enzyme] = 5 μg/mL (WT) or 25 μg/mL (mutants); [Tris-HCl] = 50 mM, pH 7.5; [glycine] = 20 mM; [benzoyl-CoA] = 100 μM; T = 25 °C.

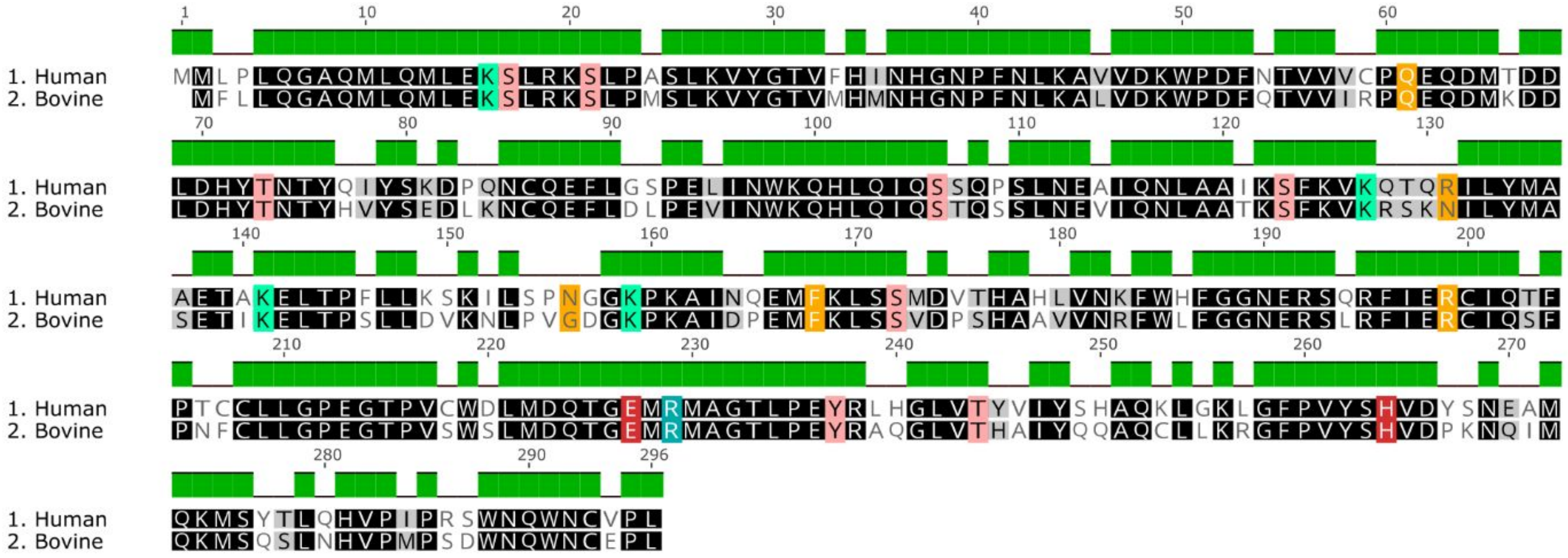

Figure S7. Alignment of the bovine and human GLYAT sequences. Identical residues are highlighted black (76% pairwise identity) and similar residues in grey (83% similarity). Important residues are marked in the alignment. Known SNPs/mutants in human GLYAT are highlighted orange, the catalytic-dyad red, and putative glycine binding residue (Arg228) in blue. Putative lysine acetylation sites are highlighted green and phosphorylation sites pink. Posttranslational modifications were predicted using the online tool GPS-PAIL[13] and GPS 5.0[14], respectively.

REFERENCES

(1) Sanchis, J.; Fernández, L.; Carballeira, J. D.; Drone, J.; Gumulya, Y.; Höbenreich, H.; Kahakeaw, D.; Kille, S.; Lohmer, R.; Peyralans, J. J. P.; Podtetenieff, J.; Prasad, S.; Soni, P.; Taglieber, A.; Wu, S.; Zilly, F. E.; Reetz, M. T. Improved PCR Method for the Creation of Saturation Mutagenesis Libraries in Directed Evolution: Application to Difficult-to-Amplify Templates. *Appl Microbiol Biotechnol* **2008**, 81 (2), 387–397. https://doi.org/10.1007/s00253-008-1678-9.

(2) Studier, F. W. Protein Production by Auto-Induction in High Density Shaking Cultures. *Protein Expr Purif* **2005**, 41 (1), 207–234. https://doi.org/10.1016/j.pep.2005.01.016.

(3) Badenhorst, C. P. S.; Jooste, M.; Van Dijk, A. A. Enzymatic Characterization and Elucidation of the Catalytic Mechanism of a Recombinant Bovine Glycine N-Acyltransferase. *Drug Metab Dispos* **2012**, 40 (2), 346–352. https://doi.org/10.1124/dmd.111.041657.

(4) Kabsch, W. XDS. *Acta Crystallogr D Biol Crystallogr* **2010**, 66 (2), 125–132. https://doi.org/10.1107/S0907444909047337.

(5) Winter, G. Xia2: An Expert System for Macromolecular Crystallography Data Reduction. *J Appl Crystallogr* **2010**, 43 (1), 186–190. https://doi.org/10.1107/S0021889809045701.

(6) Evans, P. Scaling and Assessment of Data Quality. *Acta Crystallogr D Biol Crystallogr* **2006**, 62 (1), 72–82. https://doi.org/10.1107/S0907444905036693.

(7) Usón, I.; Sheldrick, G. M. An Introduction to Experimental Phasing of Macromolecules Illustrated by SHELX; New Autotracing Features*. Acta Crystallogr D Struct Biol* **2018**, 74, 106–116. https://doi.org/10.1107/S2059798317015121.

(8) Pannu, N. S.; Waterreus, W. J.; Skubák, P.; Sikharulidze, I.; Abrahams, J. P.; De Graaff, R. A. G. Recent Advances in the CRANK Software Suite for Experimental Phasing. Acta Crystallogr D Biol Crystallogr 2011, 67 (4), 331–337. https://doi.org/10.1107/S0907444910052224

(9) Baek, M.; DiMaio, F.; Anishchenko, I.; Dauparas, J.; Ovchinnikov, S.; Lee, G. R.; Wang, J.; Cong, Q.; Kinch, L. N.; Dustin Schaeffer, R.; Millán, C.; Park, H.; Adams, C.; Glassman, C. R.; DeGiovanni, A.; Pereira, J. H.; Rodrigues, A. V.; Van Dijk, A. A.; Ebrecht, A. C.; Opperman, D. J.; Sagmeister, T.; Buhlheller, C.; Pavkov-Keller, T.; Rathinaswamy, M. K.; Dalwadi, U.; Yip, C. K.; Burke, J. E.; Christopher Garcia, K.; Grishin, N. V.; Adams, P. D.; Read, R. J.; Baker, D. Accurate Prediction of Protein Structures and Interactions Using a Three-Track Neural Network. *Science* **2021**, *373* (6557), 871–876. https://doi.org/10.1126/science.abj8754.

(10) McCoy, A. J.; Grosse-Kunstleve, R. W.; Adams, P. D.; Winn, M. D.; Storoni, L. C.; Read, R. J. Phaser Crystallographic Software. *J Appl Crystallogr* **2007**, 40 (4), 658–674. https://doi.org/10.1107/S0021889807021206.

(11) Emsley, P.; Lohkamp, B.; Scott, W. G.; Cowtan, K. Features and Development of Coot. *Acta Crystallogr D Biol Crystallogr* **2010**, 66 (4), 486–501. https://doi.org/10.1107/S0907444910007493.

(12) Murshudov, G. N.; Skubák, P.; Lebedev, A. A.; Pannu, N. S.; Steiner, R. A.; Nicholls, R. A.; Winn, M. D.; Long, F.; Vagin, A. A. REFMAC5 for the Refinement of Macromolecular Crystal Structures. *Acta Crystallogr D Biol Crystallogr* **2011**, 67 (4), 355–367. https://doi.org/10.1107/S0907444911001314.

(13) Deng, W., Wang, C., Zhang, Y. *et al.* GPS-PAIL: Prediction of Lysine Acetyltransferase-Specific Modification Sites from Protein Sequences. *Sci Rep* **2016**, 6, 39787. DOI: 10.1038/srep39787

(14) Wang C, Xu H, Lin S, Deng W, Zhou J, Zhang Y, Shi Y, Peng D, Xue Y. GPS 5.0: An Update on the Prediction of Kinase-specific Phosphorylation Sites in Proteins. *Genomics, Proteomics Bioinf* **2020**, 18(1):72-80. DOI: 10.1016/j.gpb.2020.01.001
